# Characteristics of and User Engagement With Antivaping Posts on Instagram: Observational Study

**DOI:** 10.2196/29600

**Published:** 2021-11-25

**Authors:** Yankun Gao, Zidian Xie, Li Sun, Chenliang Xu, Dongmei Li

**Affiliations:** 1 Department of Clinical & Translational Research University of Rochester Medical Center Rochester, NY United States; 2 Department of Computer Science University of Rochester Rochester, NY United States

**Keywords:** anti-vaping, Instagram, user engagement, e-cigarettes, vaping, social media, content analysis, public health, lung health

## Abstract

**Background:**

Although government agencies acknowledge that messages about the adverse health effects of e-cigarette use should be promoted on social media, effectively delivering those health messages is challenging. Instagram is one of the most popular social media platforms among US youth and young adults, and it has been used to educate the public about the potential harm of vaping through antivaping posts.

**Objective:**

We aim to analyze the characteristics of and user engagement with antivaping posts on Instagram to inform future message development and information delivery.

**Methods:**

A total of 11,322 Instagram posts were collected from November 18, 2019, to January 2, 2020, by using antivaping hashtags including #novape, #novaping, #stopvaping, #dontvape, #antivaping, #quitvaping, #antivape, #stopjuuling, #dontvapeonthepizza, and #escapethevape. Among those posts, 1025 posts were randomly selected and 500 antivaping posts were further identified by hand coding. The image type, image content, and account type of antivaping posts were hand coded, the text information in the caption was explored by topic modeling, and the user engagement of each category was compared.

**Results:**

Analyses found that antivaping images of the *educational/warning* type were the most common (253/500; 50.6%). The average likes of the *educational/warning* type (15 likes/post) were significantly lower than the *catchphrase* image type (these emphasized a slogan such as “athletesdontvape” in the image; 32.5 likes/post; *P*<.001). The majority of the antivaping posts contained the image content element *text* (n=332, 66.4%), followed by the image content element *people/person* (n=110, 22%). The images containing *people/person* elements (32.8 likes/post) had more likes than the images containing other elements (13.8-21.1 likes/post). The captions of the antivaping Instagram posts covered topics including “lung health,” “teen vaping,” “stop vaping,” and “vaping death cases.” Among the 500 antivaping Instagram posts, while most posts were from the *antivaping community* (n=177, 35.4%) and *personal* account types (n=182, 36.4%), the *antivaping community* account type had the highest average number of posts (1.69 posts/account). However, there was no difference in the number of likes among different account types.

**Conclusions:**

Multiple features of antivaping Instagram posts may be related to user engagement and perception. This study identified the critical elements associated with high user engagement, which could be used to design antivaping posts to deliver health-related information more efficiently.

## Introduction

Around 2006, electronic cigarettes (e-cigarettes) became commercially available in the United States [1]. Since then, the prevalence of e-cigarette use (vaping) kept increasing, particularly among youth [2,3]. Due to the short history of e-cigarettes in the market, the long-term health effects of e-cigarette use are not well known [4]. However, multiple studies have shown an association between e-cigarette use and both physical and mental disorders [5-9]. In addition, more than 2000 e-cigarette or vaping product use–associated lung injury (EVALI) cases in the United States have been reported to the CDC since August 2019 [10].

The use of the internet to analyze, detect, and forecast diseases and predict human behavior relating to public health topics is known as infodemiology, which has become an essential part of health informatics research [11,12]. Social media data is a widely used web-based source for infodemiology studies [13-15]. As of 2019, there were approximately 247 million US social media users, representing 79% of the US population [16]. Recognizing the popularity of social media, e-cigarette manufacturers and stores post and share content promoting e-cigarettes on social media at no cost [17-19]. They increase the dissemination reach of their products by using popular hashtags or potentially by using computer programs to generate and post e-cigarette posts automatically and frequently [20-22]. On Twitter, there have been claims of multiple benefits of e-cigarette use [18,23-30]. In addition, e-cigarette companies and vape stores also increase the popularity of their products through celebrity sponsorship or by using fake user accounts to disseminate favorable views [20,21,27]. Social bot accounts have been shown to be used for promoting e-cigarettes and touting their “health benefits” on Twitter [31].

Although there are many provaping messages on social media, there are also posts about the potential adverse health effects of e-cigarette use [20,32-38]. Exposure to e-cigarette use on social media has been shown to be associated with e-cigarette use beliefs and vaping behavior [39]. Some government agencies started to recognize the unbalanced nature of information regarding e-cigarettes on social media and identified that more discussion about the negative health effects of e-cigarette use should be promoted [40,41]. The number of Twitter accounts about quitting smoking increased from 2007-2010, and almost half of the accounts were linked to commercial sites that promote different quit smoking products [30]. Sentiment and topic analyses showed that most of the health-related posts on Twitter are antivaping [42]. On YouTube, channels that post television/internet news content discuss the dangers of e-cigarettes more frequently than channels run by consumers or e-cigarette companies [43]. The most common negative health effects of e-cigarettes mentioned on YouTube include discussions about nicotine, and known and unknown health consequences related to e-cigarette use [28]. However, Instagram, a popular social media platform used by more than half of US youth [44], has rarely been investigated in terms of its antivaping content [45]. Our previous study showed that there are fewer antivaping posts than provaping posts on Instagram and highlighted the importance of regulating e-cigarette posts on Instagram [46]. However, we have only compared the overall differences between provaping and antivaping posts on Instagram. Antivaping content has not been well studied in the context of identifying effective communication methods to inform the public about the harms of e-cigarette use.

Therefore, we downloaded Instagram images that used antivaping hashtags. We selected 500 antivaping posts and analyzed their image type, image content, text information, and account type, as well as the user engagement associated with different categories. A full understanding of antivaping Instagram posts will aid in the identification of the essential post features related to higher user engagement and awareness, and further development of high-quality messages to inform Instagram users about the health risks of e-cigarette use.

## Methods

### Data Collection

We aimed to study vaping-related content posted on Instagram before the Food and Drug Administration announced a ban on cartridges and pods with specific flavors [47]. Therefore, posts using antivaping hashtags published from November 18, 2019, to January 2, 2020, were collected through Instagram’s application programming interface. The most frequently used antivaping hashtags identified from a previous study were used to extract data; these hashtags included #novape, #novaping, #stopvaping, #dontvape, #antivaping, #quitvaping, #antivape, #stopjuuling, #dontvapeonthepizza, and #escapethevape [46]. The Instagram images and the following metadata were collected: user ID, username, post date, follower count (the number of users that follow the account), following count (the number of users that the account follows), like count, comment count, media count (the number of posts that the accounts have), picture URL, caption, and hashtags. The combination of the Instagram user ID and post date were used to remove duplicate posts. The metadata including follower count, following count, like count, and comment count were updated one month later to get more accurate information.

### Data Coding and Analysis

There were a total of 11,322 unique posts collected from Instagram during our study period from November 18, 2019, to January 2, 2020. From those posts, 1025 were randomly selected. Among the 1025 posts, 500 were antivaping posts as determined by hand coding, and these were used for further analysis. The attitude of each post toward e-cigarette use was determined by considering both image and caption content. Only the antivaping posts, which were about the potential health risks of electronic cigarette use or were against vaping behavior, were selected for further analysis. The coding of the images and their contents was similar to previous papers, with some modifications [29,46,48,49]. The posts were independently coded by two reviewers, and any differences were resolved by discussion. The reviewer agreement on classifying posts was 95.2%.

The image type, which identified the image themes, was categorized as one of the following: (1) advertisement (eg, a picture displaying discount information for quit vaping products); (2) catchphrase (eg, a picture emphasizing a slogan such as “athletesdontvape”); (3) product display (eg, a professional photo of an e-liquid container); (4) educational/warning (eg, images that state research results or facts about e-cigarettes; (5) events (eg, an image showing people attending a presentation or workshop related to e-cigarettes); (6) memes (eg, a picture created to deliver a message related to e-cigarettes while being comedic); (7) news (eg, a screenshot from a newspaper or television program of e-cigarette–related events); (8) notice (eg, a flyer about an upcoming e-cigarette–related presentation); (9) personal experience (eg, an image showing a person’s progress in quitting vaping); (10) vaping (eg, an image showing a person exhaling aerosols), and (11) others (images not falling into any previously defined category).

The content of Instagram images (ie, the objective elements shown in the images) was categorized into the following categories: (1) cartoon (as defined in the Master Settlement Agreement [19,50]); (2) text (eg, an image containing text information); (3) people/person (eg, an image with the major content of one or more people; (4) vaping (eg, an image displaying a person exhaling aerosols); (5) sign (eg, an image showing the sign of “no vaping allowed”); (6) product (eg, an image containing an e-cigarette device); and (7) others (images displaying items not falling into any category defined above). Each image might contain multiple content elements.

Since the attitudes of Instagram posts were determined based on both image and text content, the latent Dirichlet allocation (LDA) topic model was applied to the antivaping posts’ captions to analyze the text content of the antivaping posts [51]. Punctuation, stop words, and white spaces in the captions were removed to clean the data. Uppercase characters were converted to lowercase, and words were lemmatized to their stem form. Gensim (RARE Technologies Ltd) was used to identify frequent bigrams and trigrams. The optimal number of topics was determined based on topic coherence [52].

The posts were traced back to the posters’ Instagram accounts to determine the account type: (1) antivaping community (eg, a local government organization that is specifically against teens vaping); (2) personal, for example, a person who does not have either commercial (selling/promoting products) or professional (sharing professional knowledge) affiliations; (3) community (eg, a city account that uploads all their local news, which includes e-cigarette–related information), and (4) a business organization (eg, a company that promotes its essential oil products by claiming they can help with quitting e-cigarette use).

The number of likes was used to indicate the user engagement of each Instagram post. One-way analysis of variance and Tukey’s honestly significant difference (HSD) post hoc test were used to compare the means of likes for different categories of each feature, as well as the means of media_count and follower_count by using JMP Pro 15 (SAS Institute Inc). The correlations between media_count and follower_count for each account type were analyzed by Spearman correlation. Due to the large variation of real-life data, the top 5% and bottom 5% (outliers) of likes, media_count, and follower_count were removed from each category of each feature to compare the mean values [53].

## Results

### Characteristics of Antivaping Posts

Table 1 displays the distribution of the frequency of each image type. The most popular image type was *educational/warning* (253/500, 50.6%), followed by *memes* (n=36/500, 7.2%), *catchphrase* (n=35/500, 7%), *news* (n=29/500, 5.8%), *events* (n=28/500, 5.6%), and *vaping* (n=27/500, 5.4%). Further analysis compared the mean of likes among different image types (Table 1). Average numbers of likes for the *catchphrase* (mean 32.5) and *educational/warning* (mean 15) types were significantly higher than for *advertisement* posts (mean 8.2). In addition, the *others* type (mean 36.1) had significantly more likes than the *advertisement*, *vaping* (mean 15), *educational/warning*, and *notice* (mean 11.8) types.

**Table 1:** Image types of antivaping posts on Instagram (N=500).

Image type	Posts, n (%)	Mean likes (95% CI)
Advertisement	7 (1.4)	8.2 (2.8-13.6)
Catchphrase	35 (7)	32.5 (15.6-49.4)
Product display	15 (3)	18 (9.3-26.7)
Educational/warning	253 (50.6)	15 (13.6-16.4)
Events	28 (5.6)	22.6 (16.1-29.1)
Memes	36 (7.2)	18.3 (12.0-24.5)
News	29 (5.8)	19.4 (12.7-26.1)
Notice	10 (2)	11.8 (5.5-18.0)
Personal experience	9 (1.8)	25.1 (15.2-35.1)
Vaping	27 (5.4)	15 (8.9-21.0)
Others	51 (10.2)	36.1 (24.1-48.1)

To test if any image content element is associated with higher user engagement, the image content was analyzed. The analyses indicated that most of the antivaping posts contained *text* information (n=332, 66.4%), while *people/person* content appeared in 22% (n=110) of the posts. The proportions of posts containing each of the other image content elements were all close to 10% (*cartoon*: n=60, 12%; *media information*: n=45, 9%; *vaping*: n=55, 11%; *product*: n=54, 10.8%; *sign*: n=64, 12.8%; and *others*: n=53, 10.6%). Comparison of the means of likes among different image content types showed that the *people/person* content element (mean 32.8) had significantly more likes than the *cartoon* (mean 15.7), *media information* (mean 18.1), *text* (mean 16.2), *vaping* (mean 13.8), *product* (mean 19.2), *sign* (mean 15.4), and *others* (mean 21.1) elements.

Of the 500 antivaping posts, 483 contained captions. The LDA topic model was applied to those captions to reveal the content of Instagram antivaping posts. The identified popular topics were “lung health,” “teen vaping,” “stop vaping,” and “vaping death cases” (Table 2).

**Table 2:** Caption analyses of antivaping Instagram posts.

Topic category	Keywords
Lung health	lung, ita, day, dona, make, time, healthy, start, week
Teen vaping	juul, nicotine, teen, kid, flavor, tobacco, youth, addiction, danger, school
Stop vaping	vape, stopvap, smoke, vap, novap, smoking, stop, quitsmok, tobacco, quitvap
Vaping death cases	vap, cigarette, product, health, year, people, case, death, state, report

### Antivaping User Accounts

The selected 500 antivaping Instagram posts were posted by 393 unique Instagram accounts. Table 3 showed that the most popular account types were the *antivaping community* (n=177, 35.4%) and *personal* (n=182, 36.4%) account types. The rest of the posts were from *community* (n=99, 19.8%) and *business organization* (n=42, 8.4%) account types. Multiple posts might have been posted by the same Instagram account. On average, the *antivaping community* account type had the highest number of posts per account (1.69), followed by the *business organization* account type (1.31). The *community* and *personal* account types had an average number of 1.16 and 1.06 posts per account, respectively.

Statistical analyses showed that the *community* account type (mean 685) had significantly more followers than the *antivaping community* (mean 200; *P*<.001) and *personal* (mean 361.6; *P*<.001) account types. The number of followers for the *personal* account type was significantly more than for the *antivaping community* account type (*P*=.03). The *community* (mean 497.5) and *personal* (mean 361.6) account types posted significantly more images than the *business organization* (mean 145.8; *P*<.001 and *P*=.02, respectively) and *antivaping community* (mean 81.6; *P*<.001 and *P*<.001, respectively) account types. The numbers of posts by accounts and followers of the *antivaping community* (Spearman *ρ*=0.8230), *community* (Spearman *ρ*=0.7646), *business organization* (Spearman *ρ*=0.6601), and *personal* (Spearman *ρ*=0.5511) account types were all significantly correlated (all *P*<.001). However, no significant difference was observed in the mean number of likes across account types.

**Table 3:** Account type analyses of antivaping posts on Instagram.

Analyses	Community	Antivaping community	Personal	Business organization
Number of posts (N=500)	99	177	182	42
Percentage, %	19.8	35.4	36.4	8.4
Number of accounts (N=393)	85	105	171	32
Posts/account (average of 1.27 across all types)	1.16	1.69	1.06	1.31
Mean followers (95% CI)	685 (504.2-865.8)	200 (153.9-246.1)	364 (311.5-416.5)	354.9 (172.8-537.0)
Mean media (95% CI)	497.5 (365.0-630.0)	81.6 (60.4-102.8)	361.6 (304.6-418.5)	145.8 (76.0-215.5)
Correlation of followers and media (*P* value)	0.7646 (<.001)	0.8230 (<.001)	0.5511 (<.001)	0.6601 (<.001)
Mean likes (95% CI)	16.2 (12.7-19.6)	17.4 (14.9-20.0)	20.7 (18.1-23.3)	15.3 (11.7-19.0)

## Discussion

### Principal Findings

In this study, we found that the *educational/warning* antivaping Instagram images were the most common images, while the *catchphrase* images had the highest average number of “likes.” Within different types of image content, the most popular element was *text*, while the *people/person* element had the most user engagement. The topics covered by the antivaping posts’ captions included “lung health,” “teen vaping,” “stop vaping,” and “vaping death cases.” Most of the antivaping posts were from *antivaping community* and *personal* account types. However, the *antivaping community* account type had the highest average number of posts.

### Comparison With Prior Work

Although *educational/warning* images were more popular than the other types, the *catchphrase* images and the *others* group had the most “likes.” The *catchphrase* images were mainly associated with specific populations, such as athletes, parents, or high school students. These populations created slogans, which were also used as hashtags, to signal their social identity and their stance against vaping, such as #athletesdontvape and #itsnotcooltojuulinschool. Previous studies have shown that some provaping hashtags were created by vapers through a folksonomy process, and vaping communities might encourage the spread of specific vaping practices [54,55]. Therefore, the self-identification of antivaping Instagram users may have contributed to the higher user engagement of *catchphrase* images, offering a potentially effective approach to engaging populations with different identities to broaden the impact of antivaping education among the public.

The *others* image type included some unconventional pictures that did not fall into any other defined categories. Some of those images were not high quality, while others had links to vaping, or had antivaping-related information presented in the captions. For example, there was one image from a *personal* account that only had a few thousand followers. However, in the caption, the user described a traumatizing experience, explaining that their lungs were collapsing due to excessive vaping, which resulted in tens of thousands of “likes” and intense discussions about the harm of vaping. Therefore, the caption seemed to be a powerful feature for engaging users, although Instagram is a visual social media platform. The captions of the 483 antivaping posts all covered topics like “lung health,” “teen vaping,” “stop vaping,” and “vaping death cases.” Understanding how to use captions as part of an effective cessation strategy is very important, and our data suggested that using a storytelling approach to share a user’s vaping experiences could be one option. These evidence-based messages could facilitate user interactions and appeal to fear, which has been recommended as a valid approach to raise awareness about health concerns [56].

Other than the image style and caption content, the impact that Instagram accounts have could also affect user interactions. There were 4 account types identified from the 500 antivaping posts. The *community* account type had the highest average numbers of followers (685) and posts (497.5). However, this account type posts images relating to various aspects of life rather than solely focusing on vaping-related information, which translated into a diluted frequency of antivaping posts (1.16 posts/account). In contrast, the *antivaping community* account type had the highest rate of antivaping posts (1.69 posts/account). However, this account type had a significantly lower number of followers, which could limit the impact of those accounts. There was no difference in the number of “likes” among the 4 different account types. One possible reason for this is that those accounts all publish posts with different image styles and caption content. Another possible reason might be that the exposure of Instagram users to antivaping posts may have been limited due to having fewer followers or infrequent posting, which may have caused the low number of “likes” observed for all account types.

### Limitations

Creating high-quality post content might help overcome the limitations of account impact. In this study, we found that, among the types of image content compared, posts with a *people/person* element had the highest user engagement. However, we only compared limited numbers of image objects from a limited number of posts. A larger sample size might uncover a different ranking of image content as it relates to user engagement, which is one limitation of our study. In the future, deep learning methods will be used for image object detection. Similarly, the text content of collected images will be explored using deep learning techniques to generate image captions specific to our antivaping posts. Due to the small sample size of this study, we could not determine if saturation was reached when we were classifying the types of images and accounts. In addition, we used the average number of “likes” to indicate user engagement [49], but this does not indicate the user’s support of vaping behavior [27]. Therefore, both the number and sentiment of comments should be analyzed to determine users’ attitudes toward antivaping posts.

### Conclusions

This study analyzed the features of antivaping Instagram posts that are related to user engagement and identified the most popular image type and the most active account type, which provided key insights into leveraging those features to develop and deliver antivaping messages efficiently on social media. Increasing the followers of antivaping accounts or encouraging accounts that already have a high impact (eg, influencers) to post antivaping information, as well as more frequent posts by public health entities, could potentially increase user engagement with antivaping posts and raise awareness about the risk of vaping among the public.

## Abbreviations

EVALI: e-cigarette or vaping product use–associated lung injury
FDA: Food and Drug Administration
LDA: latent Dirichlet allocation
NIH: National Institutes of Health
